# The Current State of Knowledge in Biological Properties of Cirsimaritin

**DOI:** 10.3390/antiox11091842

**Published:** 2022-09-19

**Authors:** Taoufiq Benali, Imane Jaouadi, Rokia Ghchime, Nasreddine El Omari, Kaoutar Harboul, Khalil Hammani, Maksim Rebezov, Mohammad Ali Shariati, Mohammad S. Mubarak, Jesus Simal-Gandara, Gokhan Zengin, Moon-Nyeo Park, Bonglee Kim, Shafi Mahmud, Learn-Han Lee, Abdelhakim Bouyahya

**Affiliations:** 1 Environment and Health Team, Polydisciplinary Faculty of Safi, Cadi Ayyad University, Sidi Bouzid B.P. 4162, Morocco; 2 Laboratory of Natural Resources and Environment, Polydisciplinary Faculty of Taza, Sidi Mohamed Ben Abdellah University, Taza-Gare, Taza B.P. 1223, Morocco; 3 Laboratory of Organic Chemistry, Catalysis and Environment, Department of Chemistry, Faculty of Sciences, Ibn Tofail University, B.P. 133, Kenitra 14000, Morocco; 4 Department of Clinical Neurophysiology, Hospital of Specialities, Rabat Institute, Ibn Sina University Hospital, Rabat 10056, Morocco; 5 Laboratory of Histology, Embryology, and Cytogenetic, Faculty of Medicine and Pharmacy, Mohammed V University, Rabat 10100, Morocco; 6 Department of Scientific Research, V. M. Gorbatov Federal Research Center for Food Systems, 109316 Moscow, Russia; 7 Biophotonics Center, Prokhorov General Physics Institute of the Russian Academy of Science, 119991 Moscow, Russia; 8 Department of Scientific Research, Russian State Agrarian University—Moscow Timiryazev Agricultural Academy, 49 Timiryazevskaya St., 127550 Moscow, Russia; 9 Department of Chemistry, The University of Jordan, Amman 11942, Jordan; 10 Nutrition and Bromatology Group, Department of Analytical Chemistry and Food Science, Faculty of Science, Universidade de Vigo, E-32004 Ourense, Spain; 11 Department of Biology, Faculty of Science, Selcuk Universtiy, 42130 Konya, Turkey; 12 College of Korean Medicine, Kyung Hee University, Hoigidong, Dongdaemungu, Seoul 02447, Korea; 13 Division of Genome Sciences and Cancer, The John Curtin School of Medical Research, and The Shine-Dalgarno Centre for RNA Innovation, The Australian National University, Canberra, ACT 2601, Australia; 14 Novel Bacteria and Drug Discovery Research Group (NBDD), Microbiome and Bioresource Research Strength (MBRS), Jeffrey Cheah School of Medicine and Health Sciences, Monash University Malaysia, Bandar Sunway 47500, Selangor Darul Ehsan, Malaysia; 15 Laboratory of Human Pathologies Biology, Department of Biology, Faculty of Sciences, Faculty of Medicine and Pharmacy, Mohammed V University in Rabat, Rabat 10106, Morocco

**Keywords:** bioactive compound, pharmacodynamic action, anticancer activity, apoptosis

## Abstract

The search for natural plant-based products as new pharmacological alternatives to treat various human pathologies has taken on great importance for researchers and research laboratories. In this context, research has intensified to extract and identify natural molecules endowed with biological effects. The objective of this study is to review the source and pharmacological properties of cirsimaritin. The identification and isolation of this flavonoid from various natural sources, including medicinal plants such as *Artemisia judaica*, *Cirsium japonicum*, *Lithocarpus dealbatus*, *Microtea debilis*, and *Ocimum sanctum*, has been carried out and verified using different spectral techniques. Biological effect investigations are carried out with a wide variety of experimental models in vitro and in vivo and laboratory techniques. The results of these research works showed the biological properties of cirsimaritin including anticancer, antimicrobial, antidiabetic, antiparasitic, antioxidant, and anti-inflammatory effects. The mechanisms involved in the multiple activities of this molecule are diverse and include sub-cellular, cellular, and molecular levels. Indeed, this bioactive induces anti-inflammatory and antiproliferative effects by inhibiting cell membrane receptors, interference with signaling pathways, and inhibiting transcriptional factors such as Nf-κB involved in cell promotion and proliferation. In the light of these results, cirsimaritin appears as a promising and viable alternative natural bioactive drug to treat many pathological conditions.

## 1. Introduction

The development of drugs from secondary metabolites of medicinal plants has been widely preoccupied by current scientific research [1,2,3,4,5]. Indeed, some plants synthetize a wide variety of remarkable molecules, such as daucosterol, carvone, pinosylvin, and chrysoeriol, with important health benefits such as anticancer, antidiabetic, antimicrobial, antiparasitic effects [1,6,7,8,9]. These natural bioactive compounds belong to different chemical classes such as phenolic acids, terpenoids, flavonoids, alkaloids, and terpenoids [10,11,12,13,14,15,16]. Moreover, flavonoids are considered as the most abundant and diverse family [17,18] with promising therapeutic benefits since they are non-toxic molecules with many biological activities including anticancer [19], inflammatory [20], antibacterial [21], antiviral [22], neuroprotective effects [23], and other activities. On the other hand, if their proprieties are properly used, these natural products could be efficient, safe, and new therapeutic agents. The exploration of these natural flavonoids can be a promising strategy to identify and develop drugs for pharmaceutical applications. The flavonoid cirsimaritin is found in many plants including, for example, *Artemisia judaica*, *Cirsium japonicum*, *Lithocarpus dealbatus*, *Microtea debilis*, and *Ocimum sanctum* [24,25,26,27]. Cirsimaritin has been reported to exert numerous biological effects including antimicrobial, anti-inflammatory and anti-proliferative properties [28,29,30,31].

Indeed, it showed antimicrobial effect against fungi and Gram-positive and Gram-negative bacteria [28,29,30]. This flavonoid has also demonstrated an anti-inflammatory potency, which is mediated by the phosphorylation of signal transducer and activator of transcription 3 (STAT3) and the inhibition of c-fos in RAW264 cells [32]. In addition, an antidiabetic benefit was attributed to cirsimaritin [31]. In TNF-α-treated FL83B mouse hepatocytes, its anti-hyperglycemia effects are linked to cirsimaritin’s ability to enhance glucose uptake. In silico, insulin secretion was increased after DPP-4 blockade by cirsimaritin Bower et al. [33].

Additionally, cirsimaritin had antiproliferative activity against human colon carcinoma (HT-29), human gastric adenocarcinoma (AGS), human osteosarcoma (SaOs-2), murine fibrosarcoma (WEHI-164) and human fetal foreskin fibroblast (HFFF-P16), MCF-7, and PC-3 cell lines. Its anticancer mechanisms involve some key cancer targets such as apoptosis, *p*-Akt, and cAMP/PKA signaling. Other pharmacological effects have also been described, including the inhibitory activity against the influenza A virus via the blockage of the NF-κB/p65 signal pathway [34], antioxidant effect [35,36], antiparasitic activity on *Entamoeba histolytica*, *Leishmania donovani*, *Plasmodium falciparum*, *Trypanosoma brucei rhodesiense*, *and Trypanosoma cruzi* [37], antinociceptive, and anxiolytic activities [38], as well as the protective effect of beta-cells against STZ. Even though several studies have highlighted the health benefits and biological properties of cirsimaritin, no reviews have been published to discuss and explore its properties. Therefore, the aim of this review was to represent the pharmacological activities of cirsimaritin by clarifying the molecular mechanisms responsible for these properties in the context to explore new pharmaceutical opportunities for this natural molecule and to provide a significant starting point for future studies.

## 2. Materials and Methods

The bibliometric research was carried out in a global way, without exclusion criteria and without inclusion criteria, from several databases (since 1963 to 2022), including PubMed, science-direct, Google-Scholar, Scopus, and Web of Sciences. Different keywords including cirsimaritin, biological properties of cirsimaritin, anticancer effects of cirsimaritin, antimicrobial effects of cirsimaritin, pharmacological properties of cirsimaritin were used to find data. The data collected were firstly classified according to different sections (sources and different biological and pharmacological properties). Then, the publications of each section were organized in tables and explored. These data were finally discussed and highlighted. The molecular structure of cirsimaritin was designed using Chem-Draw program.

## 3. Results and Discussion

### 3.1. Sources of Cirsimaritin

**Figure 1:**
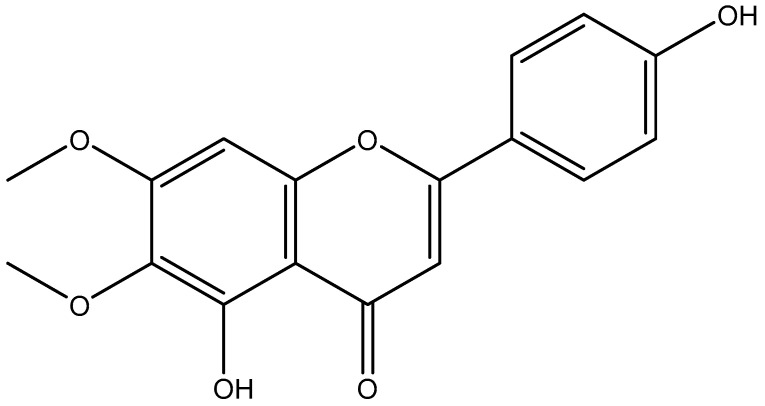
Chemical structure of Cirsimaritin (drawn by Chem-Draw).

In 1963, cirsimaritin, known as flavone, was first isolated by Morita and Shimizu [39] using *Cirsium martimum*, and many others natural sources reported in Table 1; it is a small molecular natural flavonoid (Figure 1).

### 3.2. Biological and Pharmacological Properties

**Figure 2:**
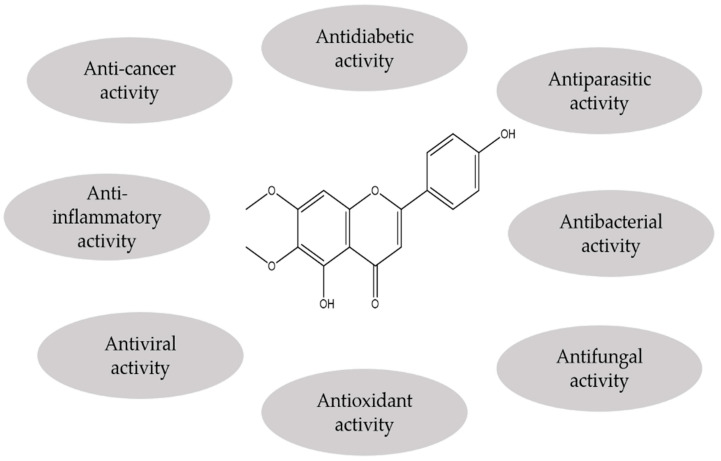
Different biological and pharmacological properties induced by cirsimaritin.

In vitro and in vivo investigations concerning cirsimaritin have shown multiple biological and pharmacological properties (Figure 2). These induced activities are due to the different mechanisms mediated by this substance. In these following sections, the pharmacological effects as well as the associated mechanisms of this compound will be described, highlighted, and discussed.

#### 3.2.1. Antibacterial and Anti-Fungal Activities

The first research reporting the antimicrobial properties of cirsimaritin dates back to 1983 (Table 2). Miski et al. [78] investigated the antibacterial effect of *Salvia palaestina* against negative and positive gram bacteria. The results showed that cirsimaritin was the only flavonoid with the highest antibacterial activity against *Escherichia coli*, *Klebsiella pneumonia*, *Pseudomonas aeruginosa*, *Proteus vulgaris*, *Staphylococcus aureus*, and *Staphylococcus epidermis* using the disk diffusion method. In another study reported by Ragasa et al. [28], cirsimaritin was found to be active against the fungi *aspergillus niger*, *Candida albicans*, and *trichophyton mentagrophytes*, and, with activity indices of 0.3, 0.3, and 0.4, respectively. However, cirsimaritin showed weak antimicrobial activity and was even inactive against *Bacillus subtilis*, *Escherichia coli*, and *Staphylococcus aureus*. Similar results were recorded by Rijo et al. [29] on the antimicrobial effect of cirsimaritin using micro-dilution and agar diffusion methods to determine Minimum Inhibitory Concentrations (MICs) and the diameter of inhibition zones, respectively. The authors showed that the most resistant pathogen to cirsimaritin was *Escherichia coli*, moreover this molecule did not inhibit *Candida albicans*, *Mycobacterium smegmatis*, and *Pseudomonas aeruginosa*, while an anti-*Staphylococcus aureus* and anti-*Enterococcus hirae* effect was observed. A study by Marino et al. [30] found that cirsimaritin, one of the major constituents of *Asphodeline anatolica* acetone extract, exhibits an antibacterial effect against *Listeria monocytogenes*, *Pseudomonas aeruginosa*, and *Staphylococcus aureus*. 

Significant antimicrobial effect of *Centaurea pseudosinaica* extract, with cirsimaritin as one of the main components, was recorded using the micro-dilution technique, against human pathogens such as *Aspergilus fumigatus*, *Candida albicans*, *Geotrichum candidum*, and *Syncephalastrum racemosum*, and Gram-positive (*Bacillis subtilis and Streptococcus pneumoniae*) and Gram-negative (*Escherichia coli* and *Pseudomonas aeruginosa*) bacteria. The results obtained clearly revealed an excellent efficacy of the alcoholic extract against all micro-organisms, especially against *Candida albicans* [51]. Interestingly, Ren and co-workers observed the antimicrobial activities of cirsimaritin identified in the bark of *Tamarix ramosissima* on *Bacillus cereus*, *Escherichia coli*, *Staphylococcus aureus*, *Listeria monocytogene*, *Pseudomonas aeruginosa*, *Salmonella typhimurium*, *and Shigella castellani* using the disk diffusion method. The inhibitory effect of the bark extract on bacterial pathogens was appreciable. *Bacillus cereus*, *Listeria monocytogenes*, and *Staphylococcus aureus* are more sensitive to the bark extract than *Escherichia coli*, *Pseudomonas aeruginosa*, *Salmonella typhimurium*, *and Shigella castellani*. Among these bacteria, *L. monocytogenes* was the most damaging bacteria with the lowest MBC value (10 mg/mL) [83].

#### 3.2.2. Antiviral and Antiparasitic Activities

Cirsimaritin was also studied for its activities against viruses. Indeed, Yan et al. [34] tested cirsimaritin on the influenza virus and its mechanism effect. A concentration-dependent reduction of viral titers, protein synthesis, and influenza A virus (IAV) RNA after cirsimaritin treatment has been demonstrated. From the point of view of the mechanism, the team suggests that the inactivation of the NF-κB/p65 signal pathway might be the origin of cirsimaritin-induced inhibition of IAV. In a computer-aided research, Hussain et al. [89] studied the cirsimaritin effect, isolated from *Santolina insularis*, on chikungunya virus (CHIKV) replication by targeting the non-structural proteins from CHIKV (nsP4, nsP3, nsP2, and nsP1). As results, cirsimaritin has shown a strong binding affinity on nsP1 compared to others.

Regarding the recent outbreaks of dangerous viruses namely COVID-19 with a rapid spread and which the development of new anti-viral drugs represents a main challenge. In this context, cirsimaritin was tested, in silico, against spike Protein SARS-CoV-2 [90]. According to this research, Cirsimaritin shows moderate binding affinity against the target protein.

Concerning parasitic infections, cirsimaritin exhibited a high inhibition versus *Plasmodium falciparum* (IC_50_ = 16.9 µM) [91], and similar activity against *Leishmania donovani*, *Trypanosoma brucei rhodesiense*, *Trypanosoma cruzi* with an IC_50_ equal of 3.9 µg/mL, 3.3 µg/mL, and 19.7 µg/mL, respectively, according to Tasdemir et al. [37]. Moreover, Quintanilla-Licea et al. [92] reported the antiprotozoal activity of this molecule against *Entamoeba histolytica* (IC_50_ = 154.26 µg/mL).

#### 3.2.3. Antioxidant Activity

Cirsimaritin represent an important benefic health molecule used in pharmaceutical industries to produce antioxidants and products against free radicals (Table 3). A study performed by Ibañez et al. [35] using DPPH assay revealed that the extracted compounds from *Rosmarinus officinalis* leaves, containing the flavonoid cirsimaritin showed high antioxidant activity. Similar results were obtained by a study investigated by Jipa et al. [36], who evaluated the antioxidant activity of *Rosmarinus officinalis* extract after γ-irradiation. It has been showed in this study that the cirsimaritin present in *R. officinalis* extract possess a good antioxidant property which enhanced by high-energy irradiation. Likewise, Cavero et al. [71] demonstrated the antioxidant efficiency of *Rosmarinus officinalis* extract on 1, 1-Diphenyl-2-picrylhydrazyl (DPPH). In this study, cirsimaritin was identified as one of the most important flavonoids in the extract, but the correlation matrix established by the authors, showed that this molecule is poorly correlated with the antioxidant activity (EC_50_ values). Furthermore, Kelm et al. [27] demonstrate using an antioxidant assay performed by analyzing the oxidation of model liposomes by fluorescence spectroscopy that cirsimaritin extracted from *Ocimum sanctum* surprisingly displayed poor antioxidant activity. Similarly in another study, Kolak et al. [93], demonstrated that cirsimaritin from *Salvia poculata* extract did not have antioxidant properties using *β*-carotene bleaching, ABTS cation radical scavenging activity and superoxide anion radical assays. Ben Sghaier et al. [86] demonstrated that cirsimaritin extracted from *Teucrium ramosissimum* showed an excellent antioxidant capacity at a Teac value of 2.04 µM using the ABTS. Lee et al. [53] studied the antioxidant effect of cirsimaritin isolated from Korean thistle *(Cirsium japonicum)* against DPPH. Results suggest that cirsimaritin showed potential reduction of DPPH free radicals with percentages of inhibition between 80% and 100% at a concentration of 100 µg/mL. The antioxidant capacity was observed to be significantly higher in the extract of *Artemisia Judaica* which is a rich source of cirsimaritin [94]. Another study by Fattahi et al. [57] evaluated the antioxidant potency of cirsimaritin identified among 13 natural populations of *Dracocephalum kotschyi* using the ferric reduction capacity of plasma (FRAP). The results indicate that the antioxidant ability of the plant extracts was mostly due to the surface flavonoids, notably the cirsimaritin flavonoid for which the concentration varies from 97.38 to 637.66 µg/g DW leading to a high antioxidant activity ranging from 203.39 to 681.27 µmol Fe^2+^/100 g DW. Burki et al., [95] found that the *Monotheca buxifolia* bark extract which contain cirsimaritin as an active compound showed significant antioxidant effect against DPPH, superoxide and hydrogen peroxide with respectively an inhibition percentage of 89.55, 82.10 and 80.55% at a concentration of 500 µg/mL. Dawé et al. [56] investigated the antioxidant effect of cirsimaritin isolated from *Combretum fragrans* extract. The authors showed that cirsimaritin presented potent DPPH radical scavenging activity with a reported IC_50_ value of 55.9 µM.

#### 3.2.4. Anti-Inflammatory Activity

**Figure 3:**
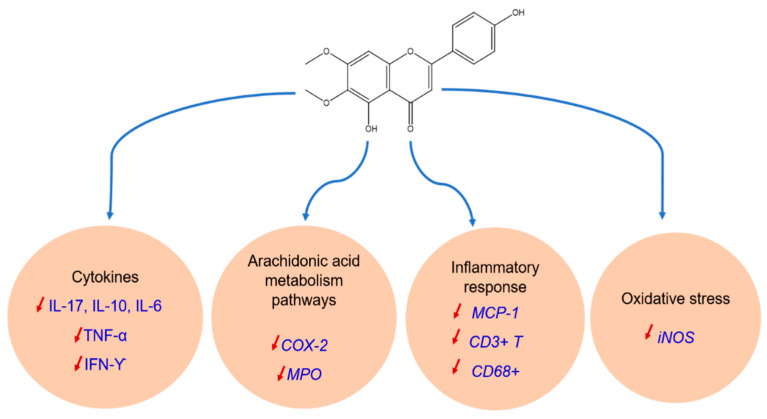
Anti-inflammatory mechanisms induced by cirsimaritin.

In a study conducted by Shin et al. [32] on the anti-inflammatory properties and mechanisms of action of cirsimaritin derived from an ethanolic extract of *Cirsium japonicum* var. *maxime maackii*, using RAW264.7 cells; the extract and cirsimaritin inhibit nitric oxide (NO) production and inducible expression of NO synthase in RAW264.7 cells. In addition, cirsimaritin can reduce inflammatory response by increasing of MCP-1, CD3+ T, CD68+ (Figure 3).

Cirsimaritin also caused the blockage of the production of different cytokines including IL-6, IL-10, IL-6, IFN-γ, and TNF-α, as well a decrease in NO production in a dose-dependent manner in RAW264.7 via the repression iNOS expression (Figure 3) [32]. It also suppressed the activation of the transcription factors induced by LPS, namely c-fos, STAT3. From these findings, this flavonoid may have an anti-inflammatory effect, which is regulated by the phosphorylation of STAT3 and the inhibition of c-fos in RAW264 cells. This inhibition of gene expression induces a decrease of cyclooxygenase-2 (COX-2) and Myeloperoxidase (MPO) (Figure 3).

Two other research works conducted separately by Al Ati et al. [38] and Cottiglia et al. [68] on cirsimaritin, isolated from traditionally used medicinal plants, showed a very significant anti-inflammatory activity. In the same context, another research study carried out by Kelm et al. [27] evaluated the cyclooxygenase (COX) inhibitory activity of cirsimaritin, at concentrations greater than 1000 µM, purified from the extract of fresh stems and leaves of *Ocimum sanctum*, while comparing with naproxen, ibuprofen and aspirin at concentrations of 10, 10, and 1000 M, respectively.

During the research performed by Kuo et al. [96], rosemary (*Rosmarinus officinalis*) extract containing cirsimaritin showed a dose-dependent effect on the expression of inflammatory mediators, in particular on lipid peroxidation. Nevertheless, the simultaneous connection of anti-inflammatory activity between rosemary extract (from SC-CO (2) at 5000 psi and 80 °C) and its pure carnosic acid (AC) via murine macrophage cells RAW 264.7 treated with LPS was determined. In addition, CA and SCCO (2) distinctly inhibited LPS-induced NO production, phosphorylated IkappaB (P-IκB), TNF-α, and NF-κB/p65 the inducible nitric oxide synthase (iNOS) and the expression of COX-2 (Figure 3). From these data, it can be concluded that cirsimaritin exhibits a significant anti-inflammatory effect with better inhibitory activity on NO (IC_50_ of 22.5 M or 7.47 g/mL) in comparison with the SCCO extract (IC_50_ of 14.50 g/mL).

#### 3.2.5. Antidiabetic Activity

**Figure 4:**
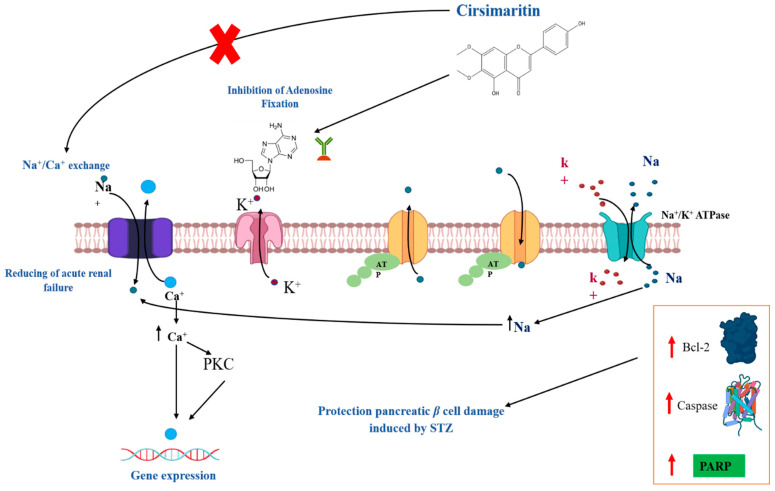
Antidiabetic mechanisms of cirsimaritin.

Numerous studies have revealed the therapeutic potential of cirsimaritin in the treatment of diabetes. Research conducted by Stefkov et al. [85] investigated the biochemical mechanism of the insulinotropic and antihyperglycemic effects of *T. polium* extracts containing cirsimaritin. Therefore, an interesting insulinotropic effect on INS1E cells at a dose of 500 µg/mL is observed. After 8 h following administration, the doses of 125 mg/kg (the same doses of the extract) administered intra-gastrointestinally in hyperglycemic and normal rats were found to be more effective in lowering blood glucose compared to intraperitoneal injection (35% reduction vs. 24%) with a large effect (50% reduction). After a week and a half of treatment, the comparison of the effect levels following the administration of glibenclamide at 2.5 mg/kg (reduction of 38%), demonstrates the absence of effects on the blood lipid profiles. As for the oral glucose tolerance assay, the extract lowered blood sugar by about 35%. Consequently, it was found that treatment results in a decrease in hepatic glycogen and tends to normalize the effect of gluconeogenesis enzymes. In another study performed with virtual screening, cirsimaritin has good binding affinities for DPP-4 and, once DPP-4 will be blocked, insulin secretion will increase (Figure 4) [33]. Moreover, by facilitating of Na/Ca exchange, cirsimaritin can modulate gene expression of several intracellular proteins involved in glucose metabolism regulation including PKC. It can also increase the expression of key factors involved in apoptosis preventing thus the pancreatic β cells from apoptotic depending on glucotoxicity and/or lipotoxicity (Figure 4).

In TNF-α-treated mouse FL83B hepatocytes, Xu et al. [73] investigated the mechanism by which the bioactive fractions from *Ruellia tuberosa* enhanced insulin resistance using a glucose uptake assay. Among them, EAF5-5 fraction, which contained syringic acid (27.3 μg/g), *p*-coumaric acid (95.0 μg/g), and cirsimaritin (805.5 μg/g) markedly improve glucose uptake. Those authors suggested that cirsimaritin may be considered as one major active ingredient from the EAF5-5 combination involved in the enhancement of glucose uptake rate of insulin-resistant FL83B hepatocytes.

#### 3.2.6. Anti-Cancer Activity

Several studies have been carried out on cell cultures showed that cirsimaritin exerts antiproliferative activities [97,98,99,100,101,102,103] on numerous cancer cell lines (Table 4). In this regard, Moghaddam et al. [97] studied, in vitro, the antiproliferative activity of cirsimaritin isolated from *Dracocephalum kotschyi* against normal and malignant cell lines using the MTT test. As results, this molecule showed a moderate inhibition of HT-29 and AGS cell lines proliferation compared to SaOs-2 and WEHI-164. Likewise, Bai et al. [63] have shown that the said substance also exhibits a moderate anti-proliferative activity on COLO-205 cells with IC_50_ values equal of 13.1 μM.

Another study released by Sen et al. [50] indicates that cirsimaritin, isolated from the chloroform extract of *Centaurea kilaea* Boiss, exhibits significant anticancer activity, especially against breast cancer, and the value (0.5–50 μg/mL) was taken versus a normal cell line (L929, mouse fibroblast) and certain human cancer cell lines (cervical carcinoma, MCF-7, prostate carcinoma, PC-3, and breast carcinoma) using the MTT test with IC_50_ value equal to 4.3 μg/mL.

**Figure 5:**
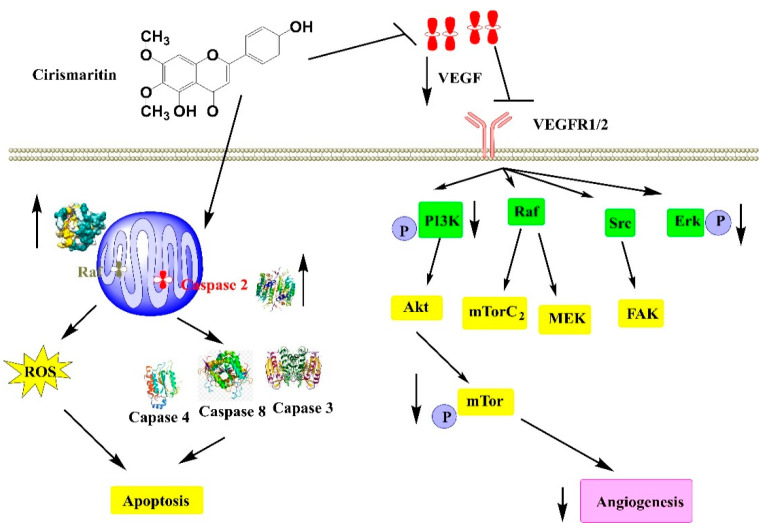
Anticancer mechanisms of cirsimaritin.

Using an in vivo cancer model, Awad et al. [101] recorded an antitumor activity of this molecule (separated from *Achillea fragrantissima* extract), characterized by the decrease in tumor size which can be explained by its antioxidant activities, confirmed by the increase in serum rate of TAC and reduction in serum TNF-α (Figure 5). Furthermore, the results of the histopathological examination demonstrated the induction of apoptosis.

The first report on the antitumor effects and the underlying mechanisms of cirsimaritin versus GBC-SD and GBCSD18H cells (gallbladder carcinoma cell lines), BGC-823 cells (gastric carcinoma cell line), SMMC-7721 cells (hepatoma cell line), SMMC-7721 cells (hepatoma cell line), and BGC-823 cells (gastric carcinoma cell line) was investigated by Quan et al. [99]. They showed that this cirsimaritin of synthetic origin exhibits an important antitumor activity and caused mitochondrial apoptosis in GBC-SD cells via activating caspase-4, -9, and -3 cascades, changing the mitochondrial membrane potential. Furthermore, cirsimaritin lead to the generation of reactive oxygen species in GBC-SD cells which triggers ER stress mitochondrial apoptotic pathways in GBC-SD cells. These molecular events induce an intrinsic apoptosis depending on caspases activation and ROS (Figure 5).

In another study, cirsimaritin as a major component of *Cirsium japonicum* var. *maackii*, exhibited an inhibition of the viability of HUVECs with a concentration-dependent manner, which showed it inhibited angiogenesis. This anticancer activity was assigned to decrease secretion of angiogenesis mediator vascular endothelial growth factor (VEGF). This inhibition induces a decrease PK3K, Raf, Src, and Erk which declined the rates of *p*-Akt and *p*-ERK in MDA-MB-231 cells, inducing thus a decrease of mTOR and causing an anti-angiogenesis effect [106] (Figure 5).

In recent study, cirsimaritin antiproliferative efficacity has been tested in human cancer cell lines including HaCaT, A431, K562, MDA-MB-231, A549, COLO-205, MCF-7, HaCaT, K562, NCIH- 520, and PC-3, and normal cell lines HEK 293, L132, and WRL-68 and in primary macrophages, by Pathak et al. [98]. It was found that cirsimaritin showed selective antitumor effect against NCIH-520 cell-line with IC_50_ = 23.29 μM, via increasing the apoptosis (10 and 100 μM). Moreover, cirsimaritin also inhibits the action of CATD and ODC which is responsible for the development stage of the cancer cells. In addition to this, follow Lipinski’s rule of five, it exerted a good binding score with the selected targets and it non-mutagenic.

Similarly, cirsimaritin from *Betula pubescens* and *Betula pendula* also had a role in lowering the viability, proliferation and clonogenicity of liver (HepG2), colon (DLD-1) and colon (DLD-1), and gastric (AGS) cancer cells. This flavonoid activated intrinsic caspases (3, 7, 8 and 9) mediated apoptosis [49].

#### 3.2.7. Other Biological Activities

Many biological activities can be linked with the use of cirsimaritin. Indeed, cirsimaritin inhibits the amplitude of the phasic contractions of guinea-pig ileum [108]. Furthermore, Hasrat et al. [26] demonstrated that cirsimaritin induces an adenosine antagonistic effect in rats. These results suggest that the interaction between the cirsimaritin and adenosine receptor could result in inhibition of calcium and sodium transport. According to these researchers, this flavonoid-molecule could have a beneficial effect in acute renal failure.

Similarly, Abdelhalim et al. [70] reported that cirsimaritin exerted significant antinociceptive and anxiolytic effects which the anxiolytic activity may be mediated via GABAA receptor, without any signs of acute toxicity (50–200 mg/kg). The anxiolytic-like activity cirsimaritin in mice was also reported by González-Trujano et al. [109]. At a concentration of 3 mg/Kg (i.p), the number of head-dips was decreased by this substance and the lapse of open arms was increased in the hole-board and in the plus-maze tests, respectively. Furthermore, Wang et al. [110] studied the inhibition of cirsimaritin on a formyl-methionyl-leucyl-phenylalanine-(fMLP) excited respiratory burst in rat neutrophils. Those researchers observe that it is probably that cirsimaritin inhibits the fMLP-caused respiratory burst in vivo via the closure of the phos-pholipase D (PLD) signalling pathway.

The protective effect of cirsimaritin from Korean thistle on apoptosis induced by STZ is documented by Lee et al. [103]. Their results, in pancreatic b cells, proved that cirsimaritin potentially removed apoptosis via increasing anti-apoptotic BCL-2 protein expression and reducing the activation of both caspases 8 and 3, BID and the DNA repair protein poly (ADP-ribose) polymerase (PARP).

Earlier study reported by Kim et al. [102] on the hyperpigmentation activity of cirsimaritin (isolated from *Lithocarpus dealbatus* branches) and explained the mechanism by which cirsimaritin stimulate melanogenesis in B16F10 cells (murine melanoma cells). The author’s findings indicate that this molecule stimulates the expression of tyrosinase by activation of cAMP/PKA signaling, subsequent phosphorylation of CREB, tyrosinase, TRP1 expression, and upregulation of MITF which further triggers melanogenesis.

## 4. Conclusions and Perspectives

Natural compounds have considerable benefits when evaluated for their biological effects. In this order, these molecules have been studied with a view to develop new therapeutic options to treat human diseases. According to the present review, many chemical analysis techniques revealed the presence of this flavonoid in a variety of pharmacologically active plants including *Artemisia judaica*, *Cirsium japonicum*, *Lithocarpus dealbatus*, *Microtea debilis*, and other species. In addition, different pharmacological and biological properties were investigated and the results showed that cirsimaritin has: a broad spectrum of action on a variety of pathogenic microorganisms in humans (Gram-positive and -negative bacteria, and fungi); anticancer effects against human colon carcinoma (HT-29), human gastric adenocarcinoma (AGS), human osteosarcoma (SaOs-2), murine fibrosarcoma (WEHI-164) and human fetal foreskin fibroblast (HFFF-P16), MCF-7, and PC-3 cell lines; and antidiabetic, antiparasitic, antioxidant, and anti-inflammatory effects. The mechanisms of action of cirsimaritin are not well understood, but it seems, according to the research highlighted, that this compound can exhibits its effects at different levels as it has been described above. Furthermore, further pharmacodynamic investigations should be conducted on cirsimaritin aimed at determining of its exact mode of action. Moreover, pharmacokinetic studies should also be conducted, on the one hand, to validate its: absorption, availability, metabolism, and elimination; on the other hand, to validate its safety via toxicological tests. Moreover, other perspectives concerning clinical trials of cirsimaritin against chronic inflammation would be a very proposing research perspectives to develop anti-inflammatory drugs. Moreover, a combination of cirsimaritin as an anticancer agent with used drugs in chemotherapy can give important results about the potent combinatory effects.

## Figures and Tables

**Table 1:** Plant sources of cirsimaritin.

Plants	Part Used	Botanical Families	Type of Extract	Concentration/Fraction	References
*Aeollanthus rydingianus*	Aerial parts	Lamiaceae	Me_2_CO Extract	58 mg	[29]
*Artemisia annua*	Leaves Stems	Asteraceae	Methanolic extract	1.45 mg	[40]
*Artemisia capillaris*	Spikes	Asteraceae	Methanolic extract	not specified	[41]
*Artemisia hispanica*	Aerial parts	Asteraceae	Methanolic extract	not specified	[42]
*Artemisia judaica*	Leaves Stems	Asteraceae	Ethanolic extract	not specified	[25]
*Artemisia meatlanticae*	Aerial parts	Asteraceae	Ether extract	not specified	[24]
*Artemisia monosperma*	Leaves Stems	Asteraceae	Ethanolic extract	not specified	[25]
*Artemisia ordosica II*	not specified	Asteraceae	not specified	not specified	[43]
*Artemisia scoparia*	Dried inflorescence	Asteraceae	Chloroform extract	not specified	[44]
*Artemisia xanthochroa*	Epigeal parts	Asteraceae	Ethanolic extract	not specified	[45]
*Arrabidaea brachypoda*	Flowers	Bignoniaceae	Ethanolic extract	not specified	[46]
*Asphodeline anatolica*	Leaves	Liliaceae	Acetone and methanol extract	not specified	[30]
*Baccharis conferta*	Aerial parts	Asteraceae	Ethanolic extract	not specified	[47]
*Becium grandiflorum*	Leaves	Lamiaceae	Methanolic extract	not specified	[48]
*Betula pendula*	Buds	Betulaceae	Carbon dioxide supercritical extraction	3.79 mg/g	[49]
*Betula pubescens*	Buds	Betulaceae	Carbon dioxide supercritical extraction	4.21 mg/g	[49]
*Buddleja polystachya*	Aerial parts	Buddlejaceae	Ethanolic extract (cold maceration)	not specified	[38]
*Centuarea kilaea*	Aerial parts	Asteraceae	Chloroform extract	10.2 mg	[50]
*Centaurea pseudosinaica*	Entire plant (leaves, flowers, stems)	Asteraceae	Ethanolic extract	0.52 g	[51]
*Centaurea scoparia*	Aerial parts	Asteraceae	Ethanolic extract	10 mg	[52]
*Cirsium martimum*	Leaves	Asteraceae	Methanolic extract	not specified	[39]
*Cirsium japonicum*	Aerial parts Leaves	Asteraceae	Ethanolic Extract	6.24 mg/g	[32]
				6.24 mg/g	[53]
				37.13 mg/g	[54]
*Clerodendrum mandarinorum*	Root bark	Lamiaceae	Ethanolic Extract	50 mg	[55]
*Combretum fragrans*	Leaves	Combretaceae	Methanolic extract	not specified	[56]
*Dracocephalum kotschyi*	Leaves	Lamiaceae	Diethyl Ether extract	97.3–637.6 µg/g	[57]
*Eremophila lucida*	Leaves	Myoporaceae	Ethyl acetate extract	not specified	[58]
*Eriodictyon californicum*	Leaves	Hydrophyllaceae	Ethanol extract	3.85 mg	[59]
*Herba artemisiae Scopariae*	Buds	Compositae	Ethyl acetate extract (ultrasonic)	not specified	[60]
*Hyptis fasciculata*	Aerial parts	Labiatae	Chloroform and methanol extract	19 mg	[61]
*Incarvillea arguta*	not specified	Bignoniaceae	not specified	not specified	[62]
*Microtea debilis*	Whole plant Aerial parts	Microteaceae	Aqueous & ethanol extractEthanol extract	0.7 mg/mL 65 mg	[26] [63]
*Ocimum basilicum*	Tricoms	Lamiaceae	Crude protein extract with HCl	not specified	[64]
*Ocimum gratissimum*	Above-ground biomass	Lamiaceae	Clevenger apparatus	10% of the total flavonoids	[65]
*Origanum intercedens*	Leaves	Lamiaceae	Chloroform extract	not specified	[66]
*Osimum sanctum*	Leaves Stems	Labiatae	Chloroform extract	1 mg	[27]
*Perovskia abrotanoies*	Aerial parts	Lamiaceae	Methanolic extract	10 mg	[67]
*Perovskia atriplicifolia*	Leaves	Lamiaceae	Ethanolic Extract	not specified	[68]
*Praxelis clematidea*	Aerial parts	Asteraceae	Ethanolic Extract (exhaustive maceration)	not specified	[69]
*Rosmarinus officinalis*	Leaves	Lamiaceae	Ethyl acetate extract	not specified	[70]
			Subcritical extraction with water	1.72%	[35]
			Supercritical fluid extraction	0.54–17.59%	[71]
			Ethanolic extract	not specified	[36]
			Super critical fluid extraction	not specified	[72]
*Ruellia tuberosa*	Leaves & stems	Acanthaceae	Methanolic extract	805 µg/g	[73]
*Salvia apiana*	Aerial parts	Lamiaceae	Aqueous ethanolic extract	not specified	[74]
*Salvia fruticosa*	Aerial parts	Lamiaceae	Acetonic extract (Soxtec system)	not specified	[75]
			Ethyl acetate extract (Soxtec extraction)	10.4 mg/g	[76]
*Salvia officinalis*	Leaves	Lamiaceae	Methanolic extraction (ultrasonic bath)	194 mg	[77] [31]
*Salvia palaestina*	Leaves	Lamiaceae	Benzene extract (Soxhlet)	30 mg	[78]
*Santolina insularis*	Leaves	Asteraceae	Methanolic extract	6.9 mg	[79]
*Satureja khuzistanica*	Aerial parts	Lamiaceae	Ethyl acetate extraction	5 mg	[80]
*Seriphidium stenocephalum*	not specified	Asteraceae	Methanolic extract	15 mg	[81]
*Stevia satureiifolia*	Aerial parts	Asteraceae	Dichloromethane extract	1.9%	[82]
*Tamarix ramosissima*	Bark	Tamaricaceae	Ethanolic extract	13.35 µg/mg	[83]
*Tanacetum chiliophyllum*	Stems	Compositae	Ethyl acetate extract	36 mg	[84]
*Teucrium polium*	Aerial parts	Lamiaceae	Alcohol extraction	not specified	[85]
*Teucrium ramosissimum*	Leaves	Lamiaceae	Chloroformic extract	not specified	[86]
*Trollius chinensis*	Flowers	Ranunculaceae	Ethanolic extract	14 mg	[87]
*Vitex rehmannii*	Aerial parts	Verbenaceae	Acetone extract	5 mg	[88]

**Table 2:** Antibacterial and antifungal effects of cirsimaritin.

Methods Used	Strains Tested	Key Results	References
Disk diffusion assay	*Escherichia coli*	MIC = 31.25 μg/mL, MBC = 125 μg/mL	[78]
	*Klebsiella pneumonia*	MIC = 31.25 μg/mL, MBC = 125 μg/mL	
	*Pseudomonas aeruginosa*	MIC = 45 μg/mL, MBC = 90 μg/mL	
	*Proteus vulgaris*	MIC = 31.25 μg/mL, MBC = 125 μg/mL	
	*Staphylococcus aureus*	MIC = 31.25 μg/mL, MBC = 125 μg/mL	
	*Staphylococcus epidermis*	MIC = 62.5 μg/mL, MBC = 125 μg/mL	
Disk diffusion method	*Aspergillus niger*	Φ = 13 mm at 40 µg	[28]
	*Basilus subtilis*	Φ = 0 mm at 40 µg	
	*Candida albicans*	Φ = 12 mm at 40 µg	
	*Escherichia coli*	Φ = 0 mm at 40 µg	
	*Pseudomonas aeruginosa*	Φ = 13 mm at 40 µg	
	*Staphylococcus aureus*	Φ = 11 mm at 40 µg	
	*Trichophyton mentagrophytes*	Φ = 14 mm at 40 µg	
Agar diffusion method	*Candida albicans*	No activity	[29]
	*Escherichia coli*	No activity	
	*Enterococcus hirae*	Growth zone inhibition	
	*Mycobacterium smegmatis*	No activity	
	*Pseudomonas aeruginosa*	No activity	
	*Staphylococcus aureus*	Growth zone inhibition	
Micro-dilution technique	*Aspergilus fumigatus*	MIC = 1.95 μg/mL	[51]
	*Bacillus subtilis*	MIC = 0.03 μg/mL	
	*Candida albicans*	MIC = 1.95 μg/mL	
	*Escherichia coli*	MIC = 11.25 μg/mL	
	*Geotrichum candidum*	MIC = 0.48 μg/mL	
	*Pseudomonas aeruginosa*	MIC = 50.0 μg/mL	
	*Streptococcus pneumoniae*	MIC = 7.81 μg/mL	
	*Syncephalastrum racemosum*	MIC = 12.5 μg/mL	
Micro-dilution method	*Bacillus cereus*	MIC = 5 mg/mL, MBC = 20 mg/mL	[83]
	*Escherichia coli*	MIC = 10 mg/mL, MBC = 25 mg/mL	
	*Listeria monocytogenes*	MIC = 5 mg/mL, MBC = 10 mg/mL	
	*Pseudomonas aeruginosa*	MIC > 10 mg/mL, MBC = NA	
	*Salmonella typhimurium*	MIC > 10 mg/mL, MBC = NA	
	*Shigella castellani*	MIC = 5 mg/mL, MBC = 15 mg/mL	
	*Staphylococcus aureus*	MIC = 5 mg/mL, MBC = 15 mg/mL	

MIC: Minimum Inhibitory Concentrations (mg/mL or μg/mL); MBC: Minimum bactericidal Concentrations (mg/mL or μg/mL); Φ: The diameter of inhibition zones (mm); NA: No activity.

**Table 3:** Antioxidant activities of cirsimaritin.

Used Method	Key Results	References
DPPH radical scavenging activity	EC_50_ = 11.3 µg/mL	[35]
*β*-carotene bleaching, superoxide anion radical, and ABTS cation radical scavenging activity assays	No antioxidant activity	[93]
ABTS assay	TEAC (µM) = 2.04	[86]
CUPRAC assay	TEAC (µM) = 4.7	
RP (Reducing power) assay	TEAC (µM) = 0.95	
FRAP assay	TEAC (µM) = 0.625	
DPPH Scavenging activity	Inhibition efficiency (%) = 80–100 at a concentration of 100 µg/mL	[53]
FRAP assay	AC = 203.39 to 681.27 µmol Fe^2+^/100 g DW at a concentration of 97.38–637.66 µg/g DW	[57]
DPPH assay	Significantly higher capacity to detoxify oxygen radicals	[94]
DPPH scavenging	% inhibition (at 500 µg/mL) = 89.55	[95]
Superoxide scavenging	% inhibition (at 500 µg/mL) = 82.10	
Hydrogen peroxide scavenging	% inhibition (at 500 µg/mL) = 80.55	
DPPH assay	IC_50_ = 55.9 µM	[56]

**Table 4:** Anticancer activity of cirsimaritin.

Origin	Biological Model (In Vitro or In Vivo)	Experimental Approach	Results and Mechanism of Action	References
Synthetic compound	human cancer cell lines namely COLO-205, MDA-MB-231, HaCaT, K562, A431, A549, MCF-7, PC-3, NCIH- 520, normal cell lines WRL-68, HEK 293 and L132 and in primary macrophages	MTT assay Inhibitory potential and binding interaction with the selected targets were analyzed through in vitro and in silico analysis	Inhibited the growth of NCIH-520 cell-line (IC_50_ 23.29 μM) Induced apoptosis Inhibited the activity of ODC and CATD Exhibited a good binding in silico score with the selected targets and it non-mutagenic	[98]
Synthetic compound	gallbladder carcinoma cell lines GBC-SD and GBCSD18H cells, gastric carcinoma cell line BGC-823 cells, and hepatoma cell line SMMC-7721 cells	Cytotoxicity assay Cell apoptosis assay Cell mitochondrial membrane potential assay Subcellular fractionation Western blot Small interference RNA RT)-PCR Detection of intracellular ROS	Inhibited the growth of tumor cells Induced mitochondrial apoptosis in GBC-SD cells Triggered endoplasmic reticulum (ER) stress Down-regulated the phosphorylation of Akt	[99]
*Centaurea kilaea*	one normal cell line (L-929, mouse fibroblast) three human cancer cell lines (Hela, cervix carcinoma; MCF-7, breast carcinoma; PC-3, prostate carcinoma	MTT assay	Inhibited the growth of MCF-7 and PC-3	[50]
*Teucrium ramosissimum*	Ehrlich’s ascites carcinoma model in mice	(5, 10, 20 mg/kg/d, orally)	Reduced tumor weight compared to EAC-control and cisplatin groups Induced tumor cell necrosis Reduced significantly the level of TNF-α in serum	[101]
*Teucrium ramosissimum*	human chronic myelogenous K562 cells	MTT assay	Exhibited an antiproliferative effect of human cancer cells IC_50_ = 1.015 × 10^−^^7^ mol/mL	[86]
*Lithocarpus dealbatus*	Murine melanoma B16F10 cells (CRL-6415)	Cell Morphology and Cell Viability Measurement *Measurement of Cellular Tyrosinase Activity* *Melanin Content Measurement* *Western Blotting*	Stimulated melanogenesis in B16F10 cells Activated of CREB as well as upregulation of MITF and tyrosinase expression activated by cAMP signaling	[102]
*Cirsium japonicum* var. *maackii*	human breast cancer (MCF-7) cell-based	Transactivation assay Proliferative activity	Exerted beneficial effects on MCF-7 cells Increased estrogenic activity	[103]
*Plectranthus amboinicus*	Cancer P-Glycoprotein-1, Cyclin Dependent Kinase-2, and Phosphoinositide-3-Kinase receptors	In silico anticancer Test	Exhibited an important strong anti-cancer effect	[104]
*Dracocephalum kotschyi* Boiss.	AGS, HT-29, HL60, SaOs-2, WEHI-164 and HFFF-P16 cells	MTT assay	Exhibited and antiproliferative activity of malignant cells	[97]
Isolated	Human T lymphoblasts (Jurkat Clone E6-1)	Cytotoxicity experiments Flow cytometry	Induced cytotoxicity EC_50_ = 66.8 µM (24 h) EC_50_ = 44.4 µM (48 h)	[105]
*Cirsium japonicum*	Breast cancer	Cell proliferation assay Tube-formation assay Western blot analysis	Inhibited the viability of HUVECs in a dose-dependent manner Inhibited angiogenesis by downregulation of VEGF, *p*-Akt and *p*-ERK in MDA-MB-231 cells	[106]
Betula pubescens and Betula pendula	gastric (AGS), colon (DLD-1) and liver (HepG2) cancer cells	Cell viability assay DNA biosynthesis Colony formation assay Apoptosis assay Western immunoblot Immunofluorescence microscopy	Induced apoptosis Activated caspase-3, caspase-7, caspase-8 and caspase-9 expression Upregulated p53 expression	[49]
*Quercus incana*	non-small cell lung carcinoma (NCI-H460) and normal mouse fibroblast (NIH-3T3) cell lines.	mRNA extraction and qRT-PCR Colony formation assay Flow cytometry analysis Cell cycle analysis Western blot analysis	Induced antiproliferative against NIH 3T3 (IC_50_ = 26.23 ± 0.053 μM) and in NCI-H460 (IC_50_ = 38.84 ± 0.037 μM)	[107]

## Data Availability

Not applicable.
